# Carcass Traits and Meat Quality of Fat-Tailed Lambs Fed Rosemary Residues as a Part of Concentrate

**DOI:** 10.3390/ani11030655

**Published:** 2021-03-01

**Authors:** Yathreb Yagoubi, Samir Smeti, Samia Ben Saïd, Houssem Srihi, Ilyes Mekki, Mokhtar Mahouachi, Naziha Atti

**Affiliations:** 1 Laboratoire de Productions Animales et Fourragères, INRA-Tunisia, University of Carthage, rue Hédi Karray, 2049 Ariana, Tunisia; yagoubiyathreb@hotmail.fr (Y.Y.); sam_fsb@live.fr (S.S.); houssemsrihi60@gmail.com (H.S.); ilyesyassinemekki@gmail.com (I.M.); 2 Laboratoire Appui à la Durabilité des Systèmes de Production Agricole dans la Région du Nord-Ouest, ESAK, Le Kef, Tunisia, University of Jendouba, 7100 Jendouba, Tunisia; sbensaid@gmail.com (S.B.S.); taymallahmah@gmail.com (M.M.)

**Keywords:** lambs, carcass characteristics, meat quality, vitamin E, rosemary residue

## Abstract

**Simple Summary:**

This study aims to investigate the carcass and meat quality from lambs fed a dietary treatment including rosemary residues obtained after distillation as cereal substitute in concentrate knowing that cereals are the main component of concentrate. Twenty-four male lambs from local fat-tailed Barbarine breed were allocated into three groups. They received individually oat hay as roughage and as complementation standard concentrate for control group (C) and two concentrate types containing rosemary residues (RR) for the other groups. The protein source was soybean (S) for RRS group while faba bean (F, *Vicia Faba*) which is a legume was the protein source for RRF group. The results suggest a positive action of rosemary by-products in improving phenolic and tocopherol compounds given their richness in these components. In addition, growth, the non-carcass and carcass traits and the meat physical properties were not altered.

**Abstract:**

Facing climate change implications on feeds unavailability, unconventional resources are being considered with a growing interest such as aromatic plant distillation residues with a two-fold object, enhancing meat quality by increasing the antioxidant properties and reducing feed prices which are often imported though expensive. Hence, this study aims to assess the effects of rosemary distillation residues (RR) incorporation in concentrate associated to two nitrogen sources as a substitute for standard concentrate on lamb’s growth, carcass traits and meat quality. For this, 24 Barbarine male lambs (3 months old, 17.83 ± 2.6 kg body weight) were divided into three groups. All lambs received individually 600 g of oat hay as roughage and 600 g of standard concentrate for control group, 600 g of concentrate based on RR and soybean meal for RRS group and 600 g of concentrate based on RR and faba bean for RRF group. After 65 days of experiment, all lambs were slaughtered. Phenolic and tocopherol intakes were significantly higher for both RR groups compared to control (*p* < 0.05). Growth, carcass weights, dressing percentages and non-carcass component weights were unaffected by the diet (*p* > 0.05). Moreover, regional and tissular compositions and meat physical properties were similar irrespective of the diet (*p* > 0.05). All color parameters were similar among groups (*p* > 0.05). However, meat produced by lambs receiving RR-based concentrate was richer on vitamin E and polyphenol contents than control lambs (*p* < 0.05). Rosemary by-products may substitute the standard concentrate resulting in similar lamb’s growth and carcass traits, while improving meat quality by increasing vitamin E content, which could improve its antioxidant power.

## 1. Introduction

Sheep farming has always been a vital sector in the economy of many countries worldwide since historical records began. However, in recent years it has been affected by climate changes, which directly disturbs livestock health, growth and reproduction, while the indirect effects are on the shortage of productive pastures, forages and feeds [1]. Hence, the scarcity of forage and some conventional feeds accentuated by the volatilizing prices of the imported concentrate is increasingly worrying problem for breeders. Consequently, a considerable interest has been currently given to the use of the unconventional feeds such as shrubs and agro-industrial by-products as a viable alternative for enhancing animal performance [2,3,4,5]. Among the agro-industrial by-products, those of aromatic plants are used specially to improve animal product quality to meet consumer demand for safe and high-quality foods [5,6]. The improvement of meat quality depends on their richness of numerous bioactive compounds such as the phenolic compounds and vitamins that provide an antioxidant activity to reduce meat oxidation, thus, extending the meat shelf life [7,8]—especially when the use of synthetic antioxidant become rejected [9] by consumers given their toxicological consequences. Aromatic plants have been used since antiquity [10] as folk medicine and as preservatives in foods and the best known aromatic plants are rosemary, thyme, oregano and sage which are widespread in the Mediterranean area. In Tunisia, the industry of rosemary essential oil extraction generates a great amount of residues (5460 Tm/year; [11]), which could be valorized as alternative feed for livestock given their free availability. Several investigations have studied the use of rosemary residues or essential oil as additive to the basal diet of lambs or ewes [8,12,13]. On the other hand, the concentrate for fattening lambs is based on cereals (>70%) in arid and semi-arid regions [14]. These regions are marked by the low hay production and the irregular availability of cereals such as barley and corn. For this, there is a growing resort to importation of these products with increasing prices. Moreover, soybean meal is the main protein source in lamb diets which is often imported; however, it could be replaced by faba bean (*Vicia Faba*) which is a legume and a local protein source that leads to similar animal performances [15]. Therefore, the use of rosemary residues could be widespread in sheep feeding. However, research on their use at high rates is scarce. To the best of our knowledge, only one study has dealt with their use as roughage substituted with the hay [4] and there are no studies on their use as concentrate. Therefore, our hypothesis resides in enhancing Barbarine lamb’s growth, carcass and non-carcass traits and meat quality by substituting standard concentrate with two types of concentrate based on rosemary residues associated to soybean or local protein resources such as the faba bean.

## 2. Material and Methods

### 2.1. Experimental Design, Feeds and Animals

Rosemary residues (RR) were collected from a forest in the Northwest of Tunisia after essential oil extraction. Fresh rosemary leaves were harvested and mixed for essential oils extraction by hydrodistillation (10 kg of fresh plant in 50 L of distilled water) using a Clevenger-type apparatus for 5 h; the by-product of distillation, the rosemary residues, were air-dried. Then they were ground in a manufactory and mixed with the remaining ingredients to obtain two types of concentrate based on RR that substitute the standard concentrate. The protein source was soybean (S) in RRS concentrate (31% RR, 39% barley, 16% S and 11% wheat bran) and faba bean in RRF one (33% RR, 22% barley, 29% faba bean, 8% wheat bran and 5% molasses). The standard concentrate was composed by 30% corn, 20% barley, 7.5% S and 37.5% wheat bran. All concentrates contain 3% of mineral vitamin supplement (10.0% Ca, 3.5% P, 8.0% Na, 4.4% Mg, 0.4% S, 0.4% Zn, 0.2 Mn, 0.2% Fe). The Dry matter (DM) and the chemical composition (% DM) of the experimental feeds are shown in Table 1.

### 2.2. Animals and Feeding

The experiment was carried at the farm of the High School of Agriculture of Kef. Twenty-four male lambs (3-month-old, 17.83 ± 2.6 kg of body weight (BW)), from fat-tailed Barbarine breed, were divided into three groups of 8 lambs each according to BW. Animals were allocated in individual pens and had free access to fresh water during the 65 days of trial. Lambs in each group received individually 600 g of oat hay and 600 g of standard concentrate for control group (C), 600 g of concentrate based on RR and soybean meal for RRS group and 600 g of concentrate based on RR and faba bean for RRF group. Throughout this period, the amount of feed offered and what was refused the previous day was daily recorded and then the intake calculated. The lamb’s BW was recorded at the beginning of the trial, and then monitored regularly once a week prior to the morning feeding. Average daily gain (ADG) was calculated. 

### 2.3. Slaughter Procedure and Measurements

At the end of the growth trial, all lambs were transported to the abattoir of the National Institute of Agronomic Researches of Tunisia (INRAT) where they were slaughtered after 12 h fasting with only access to water. They were weighed just before slaughter (slaughter body weight (SBW)). After slaughter, internal fats (omental and mesenteric) and non-carcass components such as skin, head, feet, gastro-intestinal tract, red organs (heart, liver, lungs and trachea) were removed. All fractions of the digestive tract (reticulo-rumen + omasum (rumen), abomasum, and intestine) were weighed full then empty after hand rinsing, in order to determine the weight of digestive contents. Hot carcass weight (HCW) was recorded and then carcasses were stored at 4 °C.

### 2.4. Carcass Cutting and Dissection

The cold carcass weight (CCW) was recorded 24 h post-mortem after chilling at 4 °C. The kidneys, kidney fat, testis and the fat tail were removed from cold carcasses and weighed; then each carcass was split longitudinally into two halves. The left half was cut into 4 joints (Leg, shoulder, neck and a block composed by ribs, loin and breast (RLB)). From RLB, the *Longissimus thoracis et lumborum* (LTL) muscle was removed and sampled to determine meat quality. The shoulders were dissected to estimate the tissular composition given the shoulder composition is representative of the carcass composition [16]. The first operation in the dissection process was the removal of subcutaneous fat. Muscles were then removed singly from bones; finally, inter-muscular fat was trimmed from muscles and bones. Each tissue was weighed individually; the sum of weights of each tissue in shoulder was used for calculation of carcass composition. The carcass composition data were reported as percentages.

### 2.5. Meat Physical Properties Measurement

The pH was measured in *Longissimus thoracis et lumborum* (LTL) muscle at 1 and 24 h (ultimate pH) post-mortem using a penetrating electrode connected to a portable pH-meter (HI 99163; Hanna Instruments, Cluj-Napoca, Romania) after calibration with two buffers (7.00 and 4.01). Meat color was measured directly on the LTL muscle surface with measured area of 8 mm, standard illuminant D65 and an observer angle of 10°, 24 h post-mortem using a Minolta chroma Meter CR-400 (Konica Minolta Holdings, Japan) according to the CIE L* a* b* space (CIE, 1978) and the bloom time from carcass to carcass was the same (3 min). The lightness (L*), redness (a*) and yellowness (b*) were directly recorded while, Hue angle (H*) and Chroma (C*) were calculated as: H* = tan^−1^(b*/a*) × 57.29, expressed in degrees, and C* = a*^2^ + b*^2^. To determine water cooking loss (WCL), meat samples were weighed (Wi: initial weight) and held in plastic bags and then immersed in a water-bath at 75° and heated for 30 min until the internal temperature reached 75 °C, which was monitored with thermocouple. Then, the bags were cooled under running tap water and blotted dry with paper towels. The cooked meat was weighed again (Wf: final weight). WCL was calculated as the difference between of sample weight before and after cooking and it was expressed as a percentage: 100 × (Wi − Wf)/Wi.

### 2.6. Meat Vitamin E and Total Phenolic Content (TPC) Analyses

Vitamin E analysis was performed according to the method described by [17] using high performance liquid chromatography. Vitamin E analysis in meat samples was previously described in details in [5]. To determine the total phenolic content (TPC) in meat, the method of [18] was used with some modifications. Briefly, 1 g of ground meat was mixed with 9 mL of milli-Q water (Ultramatic GR Wasserlab, Navarra, Spain), then, 10 mL of aqueous solution of methanol (50/50; *v*/*v*) was added. The obtained solution was shaken with vortex for 5 min. After 5 min of homogenization 500 μL of Carrez I solution (Scharlau, Barcelona, Spain) was added, while vortexing for 1 min. Then, 5 mL of acetonitrile was added to the mixture, while vortexing for 5 more minutes. The tubes were left to stand for 25 min, and then centrifuged at 4000× *g* for 15 min at 4 °C. Finally, two phases were obtained; a solid one formed by protein and lipid fraction and a liquid phase. To filter the supernatant, a 0.22 μm Polytetrafluoroethylene (PTFE) filter was used in a 15 mL tube. Then, the extract obtained was used to determine the TPC. The TPC in the liquid extract were estimated using the Folin-Ciocalteu method [18]. For that, 147 µL of water milli-Q, 13 µL of Folin-Ciocalteu reagent and 125 µL of 7% Na_2_CO_3_were added to 15 µL of the extract in a micro plate. Samples were held to stand for 1.5 h in the dark. The samples’ absorbance was measured with a spectrophotometer at 750 nm (Epoch 2 Microplate Spectrophotometer, Biotek, VT, USA) and the results were expressed as μg gallic acid equivalents (GAE)/g dried sample. 

### 2.7. Calculation and Statistical Analysis

Empty body weight (EBW) was calculated as the difference between SBW and weight of digestive contents. Commercial and real dressing percentage (CDP, RDP) were calculated according to the following equations:

CDP (%) = 100 × HCW/SBW(1)

RDP (%) = 100 × CCW/EBW(2)

The effect of using rosemary residues in dietary treatment on carcass and non-carcass traits and meat quality was assessed by one-way ANOVA, using the General Linear Model (GLM) procedure of SAS (2004) [19] according to the following model:

Yij = µ + Di+ eij(3)

(Yij = j^th^ measure of the i^th^ diet; µ = overall mean; Di = effect of the i^th^ diet (C, RRF and RRS); eij = error term). 

The differences between means were compared using the Duncan’s Multiple Range Test (DMRT) and the statistical significance was defined at *p* < 0.05.

## 3. Results

### 3.1. Feed Intake and Lamb’s Growth

All concentrate types were iso-nitrogenous and all lambs consumed comparable amounts of roughages and concentrate in the proportion of 50–50%, respectively, resulting in similar final body weight (25.2 ± 2.9 kg) and similar average daily gain (114 ± 23 g). Given this result and the free availability of rosemary residues, their inclusion in concentrate reduced its cost from 850 Tunisian Dinar/Ton for control to 562 and 539 Tunisian Dinar/Ton for RRS and RRV, respectively. In addition, the α-Tocopherol intake was higher for RRS (26.74 mg/day) and RRF (21.67 mg/day) groups than control (2.04 mg/day). Additionally, the daily intake of total phenolic compounds was similar for RRF and RRS but higher than that of control lambs (1.17 and 1.24 vs. 0.67 g/day, respectively; *p* < 0.05). 

### 3.2. Carcass Weights and Dressing Percentage 

The empty body weight, hot carcass weight, cold carcass weight and both dressing percentages were similar for all groups irrespective of the lamb’s diet (Table 2). 

### 3.3. Non-Carcass Components

All non-carcass components’ weights and proportions in the EBW were unaffected by the dietary treatment (Table 3).

### 3.4. Carcass Sectional and Tissular Composition

**Figure 1:**
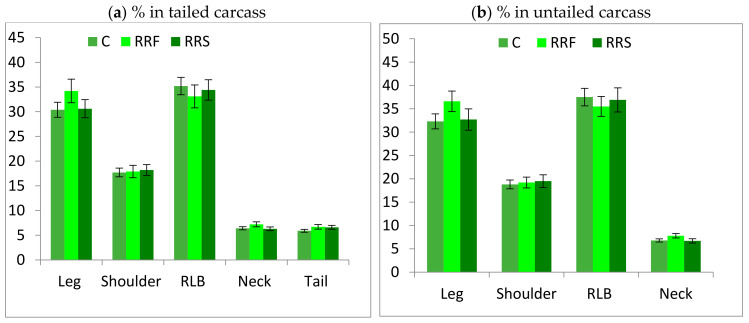
Carcass cut proportions in the tailed and untailed carcasses of Barbarine lambs fed RR-based concentrate.

**Figure 2:**
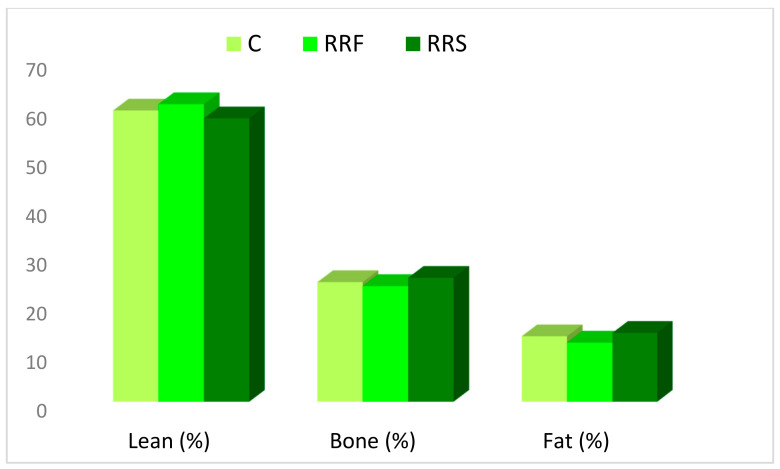
Carcass tissular proportion of Barbarine lambs fed RR-based concentrate.

The carcass sections’ weights were unaffected (*p* > 0.05) by the dietary treatments. In addition, their proportions in the tailed or untailed carcass were similar for all groups (Figure 1). The tail weight was similar for all lambs averaging 728 g and 6% of carcass weight. The substitution of standard concentrate with both types of concentrate based on RR led to the same amount and proportions of muscle, fat and bone for all groups (Figure 2). Within fat tissue in the shoulder, regardless of the diet, all lambs deposed the same amount of subcutaneous and inter-muscular fat averaging 67 and 59 g, respectively. However, the kidney fat was higher for Control (*p* < 0.05) than both RR groups (89 vs. 53 g).

### 3.5. Meat Quality

Initial pH was significantly higher for C and RRF groups compared to RRS but all the values are acceptable. After 24 h post-mortem, the ultimate pH ranged from 5.51 to 5.86 (Table 4). Although the ultimate pH of control was higher than either rosemary treatments, all values were acceptable and varied similarly given the dpH (pH24–pH1) was similar among groups (*p* > 0.05). Water cooking loss was unaffected by using RR in concentrate and was about 21.8% for all groups. All color parameters were similar among groups.

The α-Tocopherol and phenolic contents were higher (*p* < 0.05) for the meat of both experimental groups than the control one (Table 5).

## 4. Discussion

### 4.1. Feed Intake and Growth Performance

The richness of both concentrates based on RR in total phenols and tocopherol fractions (α-Tocopherol and γ-Tocopherol) was previously shown when RR were used as basal diet in lambs feeding [4]. The higher amount of these nutrients leads to higher intake of total phenolic compounds and tocopherols by lambs. In the current study, all concentrates were iso-energetic and iso-nitrogenous which explains the similarity in DM and CP intakes among groups. In contrast, when used as roughage to totally substitute oat hay at higher levels of RR incorporation (60 and 87%), the DM and CP intakes were significantly higher for groups that receive forage based on RR because they were richer on CP than oat hay [4]. The similarity in growth performances may result from the same DM intake for all treatments. Furthermore, and irrespective of concentrate type, there were no significant differences in BW gain associated with nitrogen source (soybean and faba bean), which is in line with previous reported results [20,21]. The total daily feed cost was lower for RRF and RRS than that of the control diet; hereafter, the main target of using aromatic plant by-products, the reduction of feed cost, was reached. The inclusion of RR in concentrate decreased the cost of concentrate by 36.5 and 33.8% for RRF and RRS, respectively, compared to control. Consequently, the cost/kg of gain was reduced by 40 and 22% for RRF and RRS, respectively which was 6.47, 3.88 and 5.07 Tunisian Dinar for C, RRF and RRS, respectively.

### 4.2. Carcass Weights, Dressing Percentage and Non-Carcass Components

The absence of variation for EBW, HCW, CCW as well as for commercial and real DP was generated by the similarity of SBW among all groups. This strong correlation between these parameters and SBW was previously documented [22,23,24,25]. Similar results were recorded on animal yield and carcass weights when RR were used at a low (10 to 20%) or high (60–80%) rate [4] or when ewes received myrtle by-products [26]. On the other hand, the similar concentrate proportion for all groups could be at the origin of this similarity, given that increasing dietary energy concentration or concentrate level affected significantly these parameters [27]. In addition, Asadollahi et al. [14] showed that supplementation of sugar beet pulp and roasted canola seed in a concentrate diet altered carcass traits of fattening lambs. The recorded values of CDP (44%) are lower than previous reported results for more heavy lambs of the same breed [5,26]. The dietary supplementation with aromatic plants extracts, additives or by-products had no effects on dressing percentages for sheep [4,26,28]. Regarding the nitrogen source, the substitution of soybean meal by faba bean leads to similar weights of carcasses and DP. These results are consistent with those reported in previous works where lambs received diets containing faba bean and soybean meal [21].

All lambs have similar age, sex, SBW and belonging to the same breed, which accounts for the absence of difference between dietary treatments for the non-carcass components’ weights. These facts are the main factors that influence the non-carcass components rather than intake level or diet composition [23,29,30]. The weight of offal components high in bone content (head) and/or with a low metabolic activity was similar for all lambs given these components are early maturing parts [4,23,30,31] and are less affected by dietary treatments [29]. The skin, characterized by a high metabolic activity, is related to the EBW, then, the similarity in EBW leads to similar proportions of the skin [26,32]. The similarity of the gut weight and proportion in the EBW is originated by the similarity of intake for all groups given the digestive tract weight and activity increases with feed intake [30,33]. It is well established that, in young animals, some parts of the alimentary tract and particularly the rumen continue to develop as the animals become older and heavier [21,32]. Similarly, the weights and proportions in EBW of red organs and of liver were not affected by the dietary treatments given that the liver weight did not vary under the same DM intake [30].

### 4.3. Carcass Sectional and Tissular Composition

The result on constancy of joints’ weight and proportions in the carcass confirmed the theory of anatomic harmony firstly established by [34,35] and then confirmed by other authors for fat- and thin-tailed breeds [3,4,22,24]. The dietary treatment did not affect the carcass joint’s weight and proportions and the average percentage of leg and shoulder in the tailed and untailed carcass are close to those previously reported for the same breed [4,25,26] and for other sheep from thin-tail breeds [36]. The dietary supplementation with aromatic plants by-products did not affect the carcass sectional composition as previously reported [26,32]. The carcass tissue composition depends on breed, sex, age [25] and growth rate [23], which were similar for all lambs in the current study. Moreover, the diets are iso-nitrogenous which explain the production of the same amount of muscle given a higher protein level leads to higher muscle amount [2]. In addition, the same amount of fat can be explained by the fact that lambs had the same age and the same weight. In this context, it was shown that fat depot depends on SBW, nutritional level and nutrient utilization [30,37]. The constancy of bone tissue for all groups is explained by the precocity of this tissue, which had an early development regardless of the nutrition and which depends mostly on breed and age [25,28]. The similarity of subcutaneous, inter-muscular and fat tail among groups is originated by the same SBW [32]. The subcutaneous fat deposition depends more on carcass weight than on growth level or breed [23] and occurs late, hence its proportion increased when total body fat increased while for inter-muscular fat, an early maturating depot, the inverse occurred. The similarity of tissular carcass composition irrespective of nitrogen source should encourage the use of faba bean as substitute to soybean in concentrate for growing lamb.

### 4.4. Meat Quality

The initial pH value was significantly higher for control group than experimental ones (6.33 vs. 6.18 and 5.96 for C, RRF and RRS, respectively). This trend was maintained even after 24 h with 5.86, 5.59 and 5.51 for C, RRF and RRS, respectively. The groups fed RR presented ultimate pH lower than that of control group and which are close to pH values previously reported for sheep [4,32]. However, the pH value presented by control was considered as slightly high, which could be the result of an altered utilization of dietary energy or a different reaction to the stress of slaughter [38] that leads to low muscle glycogen reserve and then a higher pH value. Furthermore, the higher phenolic compounds intake for experimental groups could probably be at the origin of this difference in pH. However, although the slight difference in ultimate pH among groups, WCL and meat color parameters were not affected and were similar among groups. In previous studies, it was shown that the intake of myrtle or rosemary essential oils as additive, or rosemary residues did affect neither the pH nor the cooking loss for lambs, ewes and goats [4,32,39]. The meat lightness (L*) values presented by all groups averaged 43.3 indicating a light-colored meat, being in the range of average acceptability of meat given a meat with lightness equal or above 34 is acceptable and close to 44 which is considered the value of acceptability by 95% of consumers [40]. Similar results were reported after a dietary intake of rosemary extracts, rosemary and myrtle by-products [4,26]. The meat redness averaged 17, which is close to the result reported in previous works showing that RR intake did not affect redness [4]. Some reports have demonstrated that natural antioxidants can retard meat color loss by extending the red color, such as oregano essential oil supplementation which increased redness (a*) and yellowness (b*) of meat [41]. In addition, similar pH and the same slaughter age are the major factors determining meat color compared to diet effect [21]. The nitrogen source did not influence the meat physical properties as previously reported [21] where, meat color, pH, WCL were similar. Meat α-Tocopherol content was significantly doubled in both groups receiving RR in concentrate compared to control. The α-Tocopherol represent the principal component of vitamin E although the presence of other tocopherols in its activity [42,43,44]. Several authors have reported the great action of diet on muscle’s tocopherol content given α-tocopherols are not degraded in the rumen but are deposited in muscle cell membranes where their antioxidant action is more effective [45,46]. Therefore, the mechanism of absorption of vitamin E is the key to maximize its benefits in meat quality [47]. Anyway, it was observed in several studies assessing the effect of ruminal microbiota and fermentation in vitamin E absorption that vitamin E was not degraded during in vitro ruminal fermentation [48]. This result is in agreement of that noted by [49], who reported that neither in vitro no in vivo hydrolysis of α-Tocopherol in the rumen, given α-Tocopherol esters need to be hydrolyzed before their absorption, little if any absorption of vitamin E in the rumen should be expected. In contrast, tocopherol esters are largely hydrolyzed in the intestinal lumen where they are then absorbed in combination with lipid micelles. Then, once in the enterocytes, vitamin E is packed into chylomicrons and delivered to the liver in the form of chylomicron remnants [50]. In the liver, the hepatic α-tocopherol transfer protein, α-TTP, binds to the vitamin E to facilitate its incorporation into nascent VLDL and its secretion from hepatocytes. This lypoprotein has a central role in vitamin E metabolism as it regulates the body-wide levels of α-tocopherol [51]. The current result is in agreement with earlier results reporting the increase of muscle’s α-tocopherol content when lambs received rosemary by-products as basal diet [5] or when sheep and goats received distillated myrtle leaves or myrtle essential oil [6,39]. The values found when animals received distillated rosemary or myrtle leaves exceed even those reported under grazing conditions although the richness of green herbs on vitamin and phenols [52,53]. The richness of meat on TPC could be explained by the richness of the diet on this component as previously shown [5,54]. In the same context, it was shown a positive transfer of phenolic compounds to lamb meat from pregnant ewes with the inclusion of rosemary by products in animal diet [54].

## 5. Conclusions 

The results provide evidence that the use of RR as cereal substitute up to 30% in concentrate for sheep feeding did not alter animal performances. This smart strategy of using aromatic plant by-products could be effective especially in the Mediterranean region, where this by-product is available in an important amount and is free. The cost per kilogram of meat produced by Barbarine lambs was reduced until 40%. In addition, the carcass quality was not altered and the meat quality was enhanced seen the use of RR rich in tocopherols and phenolic contents. On the other hand, faba bean (*Vicia Faba*) could be used as a substitute to soybean without affecting carcass nor meat quality; this nitrogen source could potentially be produced, given its production is relatively cheap compared to the nutritional value, to reduce the import of soybean meal, which is still expensive. However, future studies with greater size should verify these results.

## Figures and Tables

**Table 1:** Chemical composition of diets fed to Barbarine lambs.

Item	Oat Hay	RR	Standard Concentrate	RRF	RRS
Dry matter (%)	91.95	84.89	88.69	92.35	90.67
Crude Protein (%DM)	5.47	7.31	16.33	17.30	17.38
Organic Matter (%DM)	93.04	92.62	80.65	89.56	91.22
Neutral detergent fiber (%DM)	69.15	38.53	20.31	29.47	34.01
α-Tocopherol (µg/g DM)	4.42	217.20	0.45	52.71	62.98
γ-Tocopherol (µg/g DM)	3.97	3.78	0.78	11.85	7.38

RR: rosemary residues; RRF: rosemary residues + faba bean; RRS: rosemary residues + soybean; DM: Dry matter.

**Table 2:** Body weight, empty body weight, carcass weight and dressing percentage in Barbarine lambs fed RR-based concentrate.

Item	C	RRF	RRS	SEM	*p*
Slaughter body weight (kg)	25.75	24.25	25.12	1.91	0.67
Empty body weight(kg)	20.37	19.62	20.12	0.80	0.80
Hot carcass weight (kg)	11.28	10.61	11.01	0.54	0.71
Cold carcass weight (kg)	10.98	10.40	10.91	0.46	0.68
Commercial dressing percentage (%)	43.95	43.84	44.26	0.38	0.90
Real dressing percentage (%)	54.23	53.33	54.39	0.39	0.51

C: Control; RRF: rosemary residues + faba bean; RRS: rosemary residues + soybean; SEM: standard error mean.

**Table 3:** Fresh organ weights and proportion (%) in EBW of Barbarine lambs fed RR-based concentrate.

Organs	C	RRF	RRS	SEM	*p*
Skin (kg)	2.90	2.66	2.77	0.14	0.51
Skin (%)	14.38	13.65	13.78	0.24	0.44
Head (kg)	1.49	1.39	1.41	0.06	0.43
Head (%)	7.39	7.15	7.07	0.10	0.45
Gut (kg)	5.48	4.71	4.85	0.51	0.52
Gut (%)	26.76	24.33	24.07	1.18	0.59
Red organs (g)	874.00	749.89	719.49	0.03	0.60
Red organs (%)	4.34	3.92	3.73	0.34	0.76
Liver (g)	384.00	369.63	403.25	0.02	0.52
Liver (%)	1.90	1.90	2.02	0.05	0.63
Testis (g)	73.06	48.63	62.21	0.02	0.54
Testis (%)	0.34	0.24	0.30	0.03	0.56

C: Control; RRF: rosemary residues + faba bean; RRS: rosemary residues + soybean; SEM: standard error mean.

**Table 4:** Meat physical properties in Barbarine lambs fed RR-based concentrate.

Meat Physical Parameters	C	RRF	RRS	SEM	*p*
Initial pH	6.33 ^a^	6.18 ^ab^	5.96 ^b^	0.05	0.03
Ultimate pH	5.86 ^a^	5.59 ^b^	5.51 ^b^	0.03	0.01
dpH	−0.46	−0.59	−0.44	0.04	0.39
Water cooking loss	21.96	23.21	20.29	0.91	0.43
Lightness (L*)	43.94	42.65	43.33	0.81	0.81
Redness (a*)	17.05	16.92	16.81	0.32	0.95
Yellowness (b*)	4.05	4.39	3.04	0.27	0.13
Chroma (C*)	17.64	17.50	17.11	0.33	0.83
Hue angle (H*)	13.07 ^ab^	14.41 ^a^	10.09 ^b^	0.74	0.07

a, b: values within a row different superscript differ significantly at *p* < 0.05. C: Control; RRF: rosemary residues + faba bean; RRS: rosemary residues + soybean; SEM: standard error mean; L*: Black to White (0 to 100); a*: green to red (−60 to +60); b*: blue to yellow (−60 to +60).

**Table 5:** Meat vitamin E and total phenolic content in Barbarine lambs fed RR-based concentrate.

Item	C	RRF	RRS	SEM	*p*-Value
α-tocopherol (μg/g DM)	3.36 ^b^	6.48 ^a^	6.32 ^a^	0.12	0.001
Total phenolic content	51.33 ^b^	60.34 ^a^	60.29 ^a^	2.11	0.008

C: Control; RRF: rosemary residues + faba bean; RRS: rosemary residues + soybean; SEM: standard error mean a, b: values within a row different superscript differ significantly at *p* < 0.05.

## Data Availability

The data presented in this study are available on request from the corresponding author.
